# Role of rs9939506 polymorphism of FTO gene in resistance to eating in male adolescents

**DOI:** 10.1186/s12887-023-04310-9

**Published:** 2023-09-26

**Authors:** Ali Shaker, Soheila Shekari, Mobina Zeinalabedini, Zahra Salimi, Zahra Roumi, Khadijeh Abbasi Mobarakeh, Ali Shamsi-Goushki, Mohammad Masoumvand, Mohammad Keshavarz Mohammadian, Pegah Samani, Ghasem Azizi-Tabesh, Hanieh Shafaei, Saeid Doaei, Naser Kalantari, Maryam Gholamalizadeh

**Affiliations:** 1 Department of Cellular and Molecular Biology, Mashhad Branch, Islamic Azad Universityof Mashhad, Mashhad, Iran; 2 Department of Nutrition, Science and Research Branch, Islamic Azad University, Tehran, Iran; 3 Department of Cellular and Molecular Nutrition, School of Nutritional Sciences and Dietetics, Tehran University of Medical Sciences, Tehran, Iran; 4 Nutrition and Metabolic Diseases Research Center, Ahvaz Jundishapur University of Medical Sciences, Ahvaz, Iran; 5 Food Security Research Center, Department of Community Nutrition, School of Nutrition and Food Science, Isfahan University of Medical Sciences, Isfahan, Iran; 6 Department of Nutrition, School of Medicine, Mashhad University of Medical Sciences, Mashhad, Iran; 7 Department of Nutrition, Faculty of Medicine, Mashhad University of Medical Sciences, Mashhad, Iran; 8 Genomic Research Center, Shahid Beheshti University of Medical Sciences, Tehran, Iran; 9 School of Nursing and Midwifery, Guilan University of Medical Sciences, Rasht, Iran; 10 Department of Community Nutrition, Faculty of Nutrition and Food Technology, National Nutrition and Food Technology Research Institute, Shahid Beheshti University of Medical Sciences, Tehran, Iran; 11 Cancer Research Center, Shahid Beheshti University of Medical Sciences, Tehran, Iran

**Keywords:** FTO gene, Genotype, Obesity, Appetite, Male adolescent

## Abstract

**Background:**

Single Nucleotide Polymorphisms (SNPs) of the Fat mass and obesity-associated (*FTO*) gene may be associated with obesity by regulating appetite. The present study aimed to investigate the relationship between *FTO* genotype and resistance to eating in male adolescents.

**Methods:**

The present cross-sectional study included 246 adolescent boys in Tehran, Iran, who were assessed for self-efficacy related to weight control using the Weight Efficacy Lifestyle (WEL), questionnaire, food intake using the Food Frequency Questionnaire (FFQ), physical activity using the International Physical Activity Questionnaire (IPAQ), and anthropometric indices using Bio-Impedance Analyzer (BIA). Moreover, the participants underwent genotyping for *the* rs9930506 polymorphism of the *FTO* gene, and the relationship between FTO genotype and resistance to eating was investigated using different models of multiple linear regression.

**Results:**

According to our findings, there was a significant reverse relationship between the FTO rs9930506 genotype and resistance to eating (β: -0.16, P = 0.01). Moreover, the relationship was still significant after adjusting for age, nutritional knowledge, BMI, and mother’s BMI, educational level, and occupational status.

**Conclusion:**

According to our results, the FTO genotype had a significant effect on resistance to eating and food desires. However, there is a need for further studies to evaluate the underlying mechanisms of the effects of the FTO gene on appetite and obesity.

## Introduction

The global prevalence of adolescent overweight and obesity has been increasing dramatically in recent decades [1], reaching rates of 5.6% (previously 0.7%) and 7.8% (previously 0.9%) for girls and boys aged 5–19 years old, respectively [2]. According to the World Obesity Federation in 2019, about 206 million children and adolescents of the age group of 5–19 years will be affected by obesity by 2025, and this number is expected to increase to 254 million by 2030 [3]. Studies have shown the association of adolescent obesity with several common adulthood diseases, such as diabetes, malignancies, and cardiovascular diseases [4]. Adolescent overweight is defined as having a Body Mass Index (BMI) higher than one Standard Deviation (SD) over the median of the growth reference curve for a given age by the World Health Organization (WHO), while adolescent obesity is defined as having a BMI higher than two SD over the related median [5]. Nowadays, it is agreed upon that obesity is a multifactorial disease affected by genetic factors and environment, such as lifestyle [6]. It has been reported that lack of physical activity and high-calorie foods are the main causes of obesity in adolescents. However, genetic and hormonal factors may also play a role [7].

According to a recent study, obesity has a genetic origin, which can be multi- or monogenic. Moreover, it has been shown that multigenic obesity is quite common, while monogenic causes of obesity are rare [8]. For example, the Fat mass and obesity-associated (FTO) gene plays a crucial role in obesity [9], and adolescent overweight and obesity are strongly associated with Single Nucleotide Polymorphisms (SNPs) of the FTO gene [10]. According to the Genome-Wide Association Studies (GWAS), SNPs of the FTO gene have an essential role in regulating fat mass and adipogenesis [11]. These obesity-associated SNPs may increase body weight by altering the expression of other genes, such as IRX3 and RPGRIP1L, rather than the FTO gene [12]. Also, the SNPs of the FTO gene can regulate energy intake since the carriers of the high-risk allele of FTO consume more high-calorie food, especially fats and sugars [13], and have poor eating habits and decreased satiety levels [13]. A study on overweight children showed that the rs9939609 allele of the FTO gene was associated with greater food responses, food satisfaction, emotional eating, lower satiety responses, and eating slowness [14].

Thus, the FTO genotype may influence eating behavior by regulating energy intake instead of energy expenditure [15]. Several studies have reported a relationship between the SNPs of FTO and intake of energy and macronutrients. However, others have reported incompatible results. For example, a study reported that adults with the high-risk allele consumed fewer calories and more protein [16]. Moreover, a study by Huang et al. (2014) reported that hypocaloric weight loss diets reduced the food desires and appetite of individuals carrying the obesity-prone allele of the FTO [17]. However, the exact underlying mechanism of the effect of FTO genotype on obesity is not illustrated yet. Also, few studies have examined the effects of the SNPs of the FTO gene on the appetites of adolescents. On the other hand, the prevalence of adolescent obesity has also increased in Iran. For example, it has increased from 3.9% to 2000 to 9.3% in 2016 in 10-19-year-old boys [18]. Therefore, the present study aimed to investigate the relationship between the FTO genotype and resistance to eating in Iranian male adolescents.

## Methods

### Study design and participants

The present analytical cross-sectional study included 533 adolescent boys of the age group of 12–16 years who were recruited from two high schools in District 5 in Tehran, Iran, from April 2021 to March 2022. All participants and their parents were explained about the study goals and methodology and gave written informed consent. Moreover, the boys who had not reached puberty, those with incomplete data, and those taking medications that affected body weight or appetite, such as anti-diabetic agents, antidepressants, β-blockers, oral corticosteroids, anti-migraine agents, megestrol, diethylpropion, liraglutide, naltrexone-bupropion, phendimetrazine, and phentermine, were excluded from the study. The sample size was calculated using the OpenEPI online software and the odds ratio found in a previous study [19], while the sampling was performed using randomized cluster sampling.

The collected data included age; educational level (without high school diploma, with high school diploma, academic education), employment status (with a job, without a job), and marital status of mother (married, divorced); the attitude, performance, and self-efficacy regarding weight control; nutritional knowledge; dietary intake; physical activity; and anthropometric indices of the participants and their mothers. Moreover, the awareness of the participants regarding the foods affecting weight, cholesterol-rich foods, healthy snacks and meals, and eating habits in leisure time was evaluated using a validated Knowledge, Attitudes, and Practices (KAP) survey [20]. The maximum score of this scale was 36, 28, and 64 for the subscales of knowledge, attitudes, and practices, respectively. Moreover, the participants scoring 75% or higher than the maximum score in each scale were classified as having “good awareness”, those scoring 50-75% of the maximum score were considered as having “fair awareness”, and those scoring less than 50% were considered as having “poor awareness”.

Also, 247 participants were going to give blood samples for FTO genotyping and evaluating the presence of rs9930506 polymorphism of the FTO gene. However, 4 participants were excluded due to fear of puncture, while 4 participants were excluded because the laboratory technicians could find a suitable vein for venipuncture. Finally, blood samples were taken from 238 participants.

### Resistance to eating

Resistance to eating was evaluated using a validated 20-item version of the Weight Efficacy Lifestyle (WEL) questionnaire [21], which assesses resistance to food under considerable pressure, such as social pressures and mental and physical health problems. The questionnaire includes five subscales that each scores 0–36, with a total score of 0-180 that is calculated by summing the scores of all 5 subscales. Based on previous research, the participants were classified into two groups of low score (less than 70% of total score) and high score (70% of total score or more) in the WEL questionnaire.

### Dietary intake

The participants’ average food intake was assessed using face-to-face interviews and a validated 168-item Food Frequency Questionnaire (FFQ) [22]. Moreover, energy and macronutrient intake were estimated based on a 24-hour food recall. This approach was used for two weekdays and one weekend. Also, the intake of cereals, legumes, nuts, meats, dairy products, vegetables, fruits, oils, and junk food, such as chips, salty puffs, and soda, was evaluated using the USDA guidelines.

### Physical activity assessment

The present study used the valid International Physical Activity Questionnaire (IPAQ) for assessing the level of physical activity by recording the time passed at home, doing sports, in transportation, and sitting [23]. Then, the participants were divided into three categories: low level of physical activity (less than 600 MET min per week), moderate level of physical activity (600–3000 MET min per week), and high level of physical activity (higher than 3000 MET min per week) [24].

### Anthropometric indices

The height was measured using a tape measure with an accuracy of 0.5 cm. During height assessment, the participants did not have shoes on and their head was attached to the wall. Moreover, weight was measured using a Bio-Impedance Analyzer (BIA) device (BF-511, Omron Co., Japan) with an accuracy of 50 g. Also, BMI, rate of body fat, rate of body muscle mass, and resting metabolic rate were calculated after the age, gender, and height of the participant were entered. The validity and reliability of this device in estimating body composition had been confirmed by a previous study [25]. Based on their height and BMI, the participants were categorized using z-scores provided by WHO. Moreover, the categorization based on the rates of body fat and muscle mass was conducted using the z-scores from previous studies [26].

### FTO genotyping

A total of 247 participants underwent blood sampling to assess the presence of the rs9930506 polymorphism of the FTO gene. A total of 5 cc of blood was taken from each participant by 4 laboratory technicians. Then, the samples were transferred into pre-coded Ethylenediaminetetraacetic Acid (EDTA) tubes. After each round of blood sampling, the samples collected in the freezer were transferred to the cellular and molecular laboratory.

After separating the buffy coat, Deoxyribonucleic Acid (DNA) extraction was performed using the specific kit (Gene All, South Korea). Moreover, a Nanodrop device was used to assess the level of DNA, and the related Optical Density (OD) was measured at a wavelength of 260–280 nm. The wavelength of 260 nm was used for DNA, while the wavelength of 280 nm was used for protein and cyclic compounds, including phenols. The acceptable wavelength of 260–280 nm for DNA was considered as 1.8-2. Also, the quality of the extracted DNA was evaluated using electrophoresis on agarose gel. The Gene Runner software was used to design the primers for the Polymerase Chain Reaction (PCR) reaction by referring to the dbSNP database (http://www.ncbi.nlm.nih.gov/SNP). Following PCR, the blood samples were assessed for the presence of rs9930506 polymorphism of the FTO gene using DNA sequencing.

### Statistical analysis

The qualitative and quantitative data were analyzed using the chi-square test and the independent t-test, respectively. Moreover, the relationship between FTO genotype and resistance to eating was investigated using different models of multiple linear regression. Also, data analysis was performed using the SPSS software (version 23), while the significance level was considered 0.05.

## Results

### Relationship between resistance to eating and socio-demographic factors

The relationships between resistance to eating and socio-demographic factors are presented in Table 1. According to our findings, the participants with low resistance to eating (14.15 ± 1.32 years) were significantly older than those with high resistance to eating (13.89 ± 1.03 years, p = 0.03), and those with high resistance to eating (63.05 ± 3.6) had significantly higher nutritional knowledge compared to those with low resistance to eating (64.58 ± 5.55, p = 0.01). Moreover, resistance to eating showed no significant relationship with weight, height, BMI, rate of body fat, rate of body muscle mass, and metabolic rate of the participants (p > 0.05). Also, no relationship was found between resistance to eating and maternal factors, including weight, height, BMI, marital status, educational level, and occupational status of the participants’ mothers.

**Table 1:** Relationship between resistance to eating and socio-demographic factors

	Low resistance to eating (n = 431)	High Resistance to eating (n = 102)	P-value
Age^†^ (y)	14.15 ± 1.32	13.89 ± 1.03	0.03*
Height^†^ (cm)	170.30 ± 74.30	165.23 ± 9.57	0.17
Weight^†^ (kg)	61.48 ± 15.82	61.29 ± 18.58	0.92
BMI^†^ (kg/m^2^)	22.25 ± 4.42	22.30 ± 6.36	0.94
Fat mass^†^ (%)	19.35 ± 8.20	18.93 ± 8.93	0.66
Muscle mass^†^ (%)	38.21 ± 3.60	38.07 ± 4.003	0.74
Metabolic rate^†^ (kcal/d)	1637.82 ± 217.07	1643.55 ± 230.84	81.9
Knowledge score^†^	63.05 ± 3.6	64.58 ± 5.55	0.01*
Physical activity^†^ (m/d)	1012.20 ± 1477.36	841.2626 ± 885.27	0.13
Mother’s weight^†^ (kg)	79.7979 ± 35.58	77.26 ± 9.40	0.19
Mother’s Height^†^ (cm)	165.54 ± 4.70	164.63 ± 5.29	0.11
**Mother’s marital status** ^‡^			
Married	429 (99.5%)	100 (97.1%)	0.35
Divorced	2 (0.4%)	2 (2.0%)	
**Mother’s educational level** ^‡^			
Without a high school diploma	23 (5.3%)	9 (8.7%)	0.11
With a high school Diploma	375 (86.8%)	81 (78.7%)	
Academic education	33 (7.9%)	12 (12.6%)	
**Mothers’ occupational status** ^‡^			
Without a job	384 (88.9%)	86 (83.5%)	0.13
With a job	48 (11.1%)	17 (16.5%)	
**Number of rs9930506 allele of FTO gene** ^‡^ **(n = 233)**			
1	56 (35.4%)	29 (38.7%)	0.17
2	72 (45.6%)	39 (52.0%)	
3	30 (19.0%)	7 (9.3%)	

P-values are calculated using the independent t-test and chi-square test. *Significant difference (p < 0.05). ^†^Data expressed as mean ± SD. ^‡^Data expressed as frequency (%)

### Relationship between resistance to eating and dietary intake

The relationship between resistance to eating and dietary intake is presented in Table 2. According to our findings, there was no significant relationship between resistance to eating and consumption of certain food categories (p > 0.05).

**Table 2:** Relationship between resistance to eating and dietary intake

Food groups	Low resistance to eating (n = 431)	High Resistance to eating (n = 102)	P-value
Grains (g/d)	9.37 ± 3.19	10.17 ± 5.86	0.21
Vegetables (g/d)	3.12 ± 2.06	3.61 ± 3.64	0.21
Fruits (g/d)	4.79 ± 3.00	4.75 ± 3.30	0.20
Dairies (g/d)	4.35 ± 1.40	4.75 ± 3.19	0.41
Nuts (g/d)	8.47 ± 9.36	8.40 ± 10.24	0.18
Meats (g/d)	21.99 ± 11.16	23.73 ± 20.54	0.95
Oils (g/d)	36.59 ± 14.35	35.51 ± 34.02	0.91
Junk food (g/d)	6.29 ± 2.88	7.55 ± 5.85	0.75

P-values are calculated using the independent t-test. Data is expressed as mean ± SD. *Significant difference (p < 0.05)

### Relationship between resistance to eating and FTO genotype

**Fig. 1:**
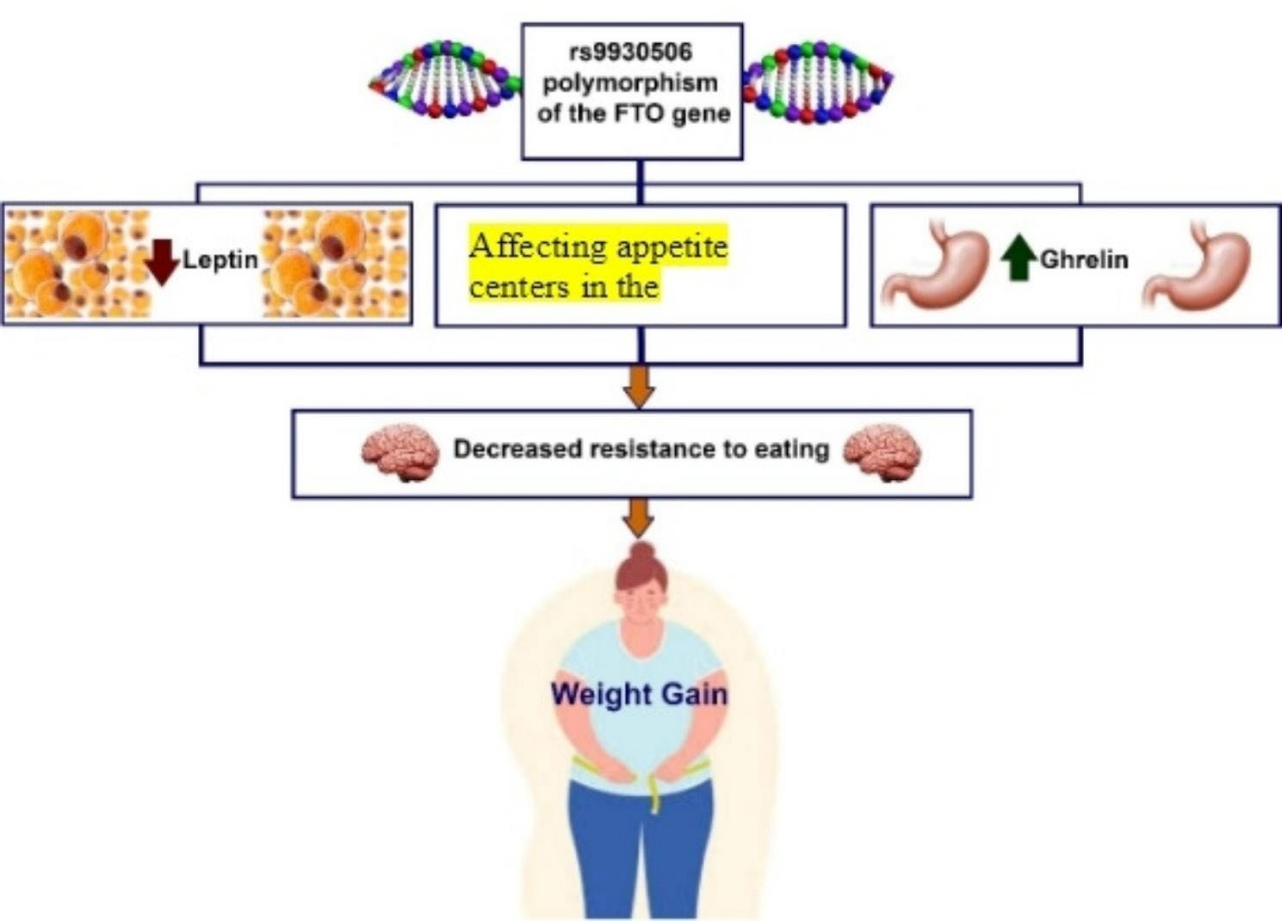
A suggested mechanism of the effect of FTO genotype on obesity

The relationship between resistance to eating and *the FTO* rs9930506 genotype is presented in Table 3. According to our findings, there was a significant reverse relationship between the *FTO* rs9930506 genotype and resistance to eating (β: -0.16, P = 0.01). Thus, the prevalence of the *FTO* rs9930506 genotype was higher in the participants with low resistance to eating. Moreover, the relationship was still significant after adjusting for age (Model 2), nutritional knowledge, and mother’s BMI, educational level, and occupational status (Model 3), as well as BMI (Model 4, Fig. 1).

**Table 3:** Relationship between resistance to eating and *FTO* rs9930506 genotype

	Model 1		Model 2		Model 3		Model 4	
	β	P	β	P	β	P	β	P
rs9930506	-0.16	0.01*	-0.16	0.01*	-0.16	0.01*	-0.15	0.02*
Model 1: Crude Model 2: adjusted for age Model 3: Model 2 + nutritional knowledge and mother’s BMI, educational level, and occupational status Model 4: Model 3 + BMI								

*Significant difference (p < 0.05)

## Discussion

The present study reported a significant reverse relationship between the FTO rs9930506 genotype and resistance to eating that remained significant after adjusting for different confounding factors. Moreover, the participants with low resistance to eating were significantly older and had significantly lower nutritional knowledge compared to those with high resistance to eating. Previous studies have shown that teenagers show more unhealthy eating behavior as they get older [27, 28]. This can be explained by the fact that older teenagers are more likely to be exposed to advertisements of unhealthy food on social networks, which decreases their self-regulation of food intake [29]. Also, studies have shown that increased nutritional knowledge helps individuals of all age groups, including teenagers, adopt healthier eating habits [30, 31].

On the other hand, the present study did not show a significant relationship between resistance to eating and consumption of certain food categories, such as grains, nuts, meats, dairy, vegetables, fruits, oils, and junk food. According to previous studies, a diet high in vegetables is associated with high satiety and less desire for sweet, salty, and fatty foods [32]. Moreover, a review showed that nuts can suppress hunger and desire to eat, leading to a feeling of fullness [33]. These findings are not compatible with ours, which can be explained by differences in data collection methods. The mentioned studies used a self-reported FFQ questionnaire for food intake assessment, which allows for potential reporting errors.

Recent studies have shown a relationship between FTO and obesity-related indices in early adolescents [34]. However, the exact mechanism of the effect of the FTO gene on body weight is not illustrated yet. According to several studies, the rs9939609 polymorphism of the FTO gene may be involved in regulating satiety and eating behaviors in children and adolescents [35, 36]. A study by Rivas et al. (2018) reported that the children with the risk allele of FTO had lower scores in satiety measures while higher scores in food responsiveness and emotional eating measures compared to other children [14], which was compatible with our findings. Moreover, a study by Ranzenhofer et al. (2019) showed a significant relationship between FTO and food intake in non-obese 5-10-year-old children whose adiposity was lower or equal to the 95th percentile [37]. Also, the studies by Emond et al. (2017) and Wardle et al. (2008) reported that children and adolescents who were carriers of the FTO risk alleles had less feeling of satiety and higher energy intake compared to others [13, 35].

Another study by Wardle et al. (2009) reported higher energy intake in children with the FTO risk alleles, which may lead to eating in the absence of hunger. Thus, carriers of the risk alleles of FTO may have low levels of eating control [36]. Moreover, several studies have investigated the effect of the rs9939609 polymorphism of the FTO gene on satiety and eating patterns in adults, reporting that the adults with the risk allele had lower levels of satiety and control on eating [38]. For example, a recent study by Melhorn et al. (2018) reported that the individuals with the risk allele had less feelings of fullness, consumed more calories, and attributed greater appeal to high-fat foods compared to other participants [38]. All these studies are compatible with our findings, suggesting that those carrying the risk allele may not manifest resistance to eating.

Several mechanisms have been suggested for the effect of the FTO gene on resistance to eating, including its effect on satiety through the Central Nervous System (CNS) and the levels of ghrelin and leptin [38, 39]. It is believed that the risk allele of the FTO gene disrupts leptin signaling through CNS, thereby increasing the size of the meals [40, 41]. This process is probably through attenuating the satiety-enhancing effect of leptin in the hindbrain or modulating mesolimbic dopamine signals [42]. Moreover, it has been shown that the rs9939609 polymorphism of the FTO gene can alter the responsiveness of CNS to cerebral ghrelin levels, possibly through mRNA expression and methylation. Such a process is a potential mediating pathway between the risk alleles of the FTO gene and obesity [17, 43].

Individuals with the rs9939609 polymorphism of the FTO are markedly different from others in neural responsiveness to food cues in cerebral regions responsible for energy homeostasis control, reward, and incentive motivation [44]. Allelic variations in the FTO gene may lead to persistent postprandial cerebral activation by visual cues of calorie-dense foods in the extended satiety network previously shown to mediate appetite, which in turn leads to increased ad libitum caloric intake [45]. Thus, those with the risk allele of the FTO gene have impaired satiety responsiveness and overconsumption. Also, Benedict et al. performed an analysis of the cross-sectional data from the Prospective Investigation of the Vasculature in Uppsala Seniors, reporting that some alleles of FTO may cause obesity by shifting the endocrine balance from leptin, the satiety hormone, to ghrelin, the hunger-promoting hormone [46]. Furthermore, another study reported that men with the risk alleles of the FTO gene had less feelings of fullness and higher levels of hunger, food cravings, appetite, and prospective food consumption [47].

The present study had some limitations as well. We conducted a cross-sectional study. Thus, the estimation of the causal relationship could not be identified. Moreover, several socioeconomic and cultural factors are involved in resistance to eating, while we only used a small sample size from two schools in Tehran. Therefore, the results of the present study cannot be generalized to other populations. Also, the present study investigated the rs9930506 polymorphism of the FTO gene. However, most other studies mentioned in the discussion section evaluated the rs9939609 polymorphism of the FTO gene. Thus, these results might not be quite comparable. On the other hand, some previous studies had used self-reported questionnaires, which increased the chance of bias. Finally, cross-sectional studies simultaneously evaluate the exposure and outcome, which gives no evidence of a temporal relationship between them.

## Conclusion

According to our results, the FTO genotype had a significant effect on resistance to eating and food desires. Moreover, there was a significant reverse relationship between the FTO rs9930506 genotype and resistance to eating. Thus, those with the FTO rs9930506 polymorphism may be at a higher risk for obesity. However, there is a need for further longitudinal studies to confirm our findings. Also, it is recommended to conduct further studies evaluating the relationship between FT genotype and intake of different micronutrients and macronutrients, as well as the underlying mechanisms of the effects of the FTO gene on appetite and obesity.

## Data Availability

The de-identified datasets generated and/or analyzed during the present study are available from the corresponding author upon reasonable request and signing of a data-sharing agreement. The original contributions presented in the study are included in the paper. Further inquiries can be directed to the corresponding author.
